# Some current problems in perovskite nano-ferroelectrics and multiferroics: kinetically-limited systems of finite lateral size

**DOI:** 10.1088/1468-6996/16/3/036001

**Published:** 2015-05-08

**Authors:** James F Scott, Alina Schilling, S E Rowley, J Marty Gregg

**Affiliations:** 1 Cavendish Laboratory, Dept. Physics, Cambridge University, Cambridge, UK; 2 Depts. of Chemistry and Physics, St. Andrews University, St. Andrews, UK; 3 Dept. Physics, Queens University, Belfast, UK; 4 CBPF, Rua Dr Xavier Sigaud 150, Urca, Rio de Janeiro, RJ 22290-180, Brazil

**Keywords:** perovskites, ferroelectrics, multiferroics

## Abstract

We describe some unsolved problems of current interest; these involve quantum critical points in ferroelectrics and problems which are not amenable to the usual density functional theory, nor to classical Landau free energy approaches (they are kinetically limited), nor even to the Landau–Kittel relationship for domain size (they do not satisfy the assumption of infinite lateral diameter) because they are dominated by finite aperiodic boundary conditions.

## 1. Introduction

Puzzles involving vertex domains in nano-ferroics of different geometries, defect-dominated dynamics, and paradoxes involving high-temperature multiferroics transcend the usual theoretical approach because in some cases (e.g., faceting and domain nucleation) they are controlled by kinetics and not thermal equilibrium—and thus the traditional Landau free-energy approach fails; or they are completely determined by aperiodic boundary conditions (faceting again! or domain widths and switching in nano-crystals) for which neither the density functional theory (DFT) cylindrical periodic boundary conditions nor the Landau–Kittel assumption of infinite lateral surface diameter is satisfied. Finally we consider the idiosyncrasies of quantum critical points (QCPs) in uniaxial ferroelectrics and multiferroics. In the quantum critical description of phase transitions the dynamics of the order parameter fluctuations affect the thermodynamic properties such as the dielectric constant below a certain characteristic temperature scale. We also note that in such cases is that although the electrocaloric coefficient diverges near *T* = 0, it is not permissible to use the Maxwell relations for indirect measurement of cooling *ΔT*, because those are entropy-based thermodynamic relationships.

### 1.1. Semantics: vortex and vertex domains: winding numbers

**Figure 1.**
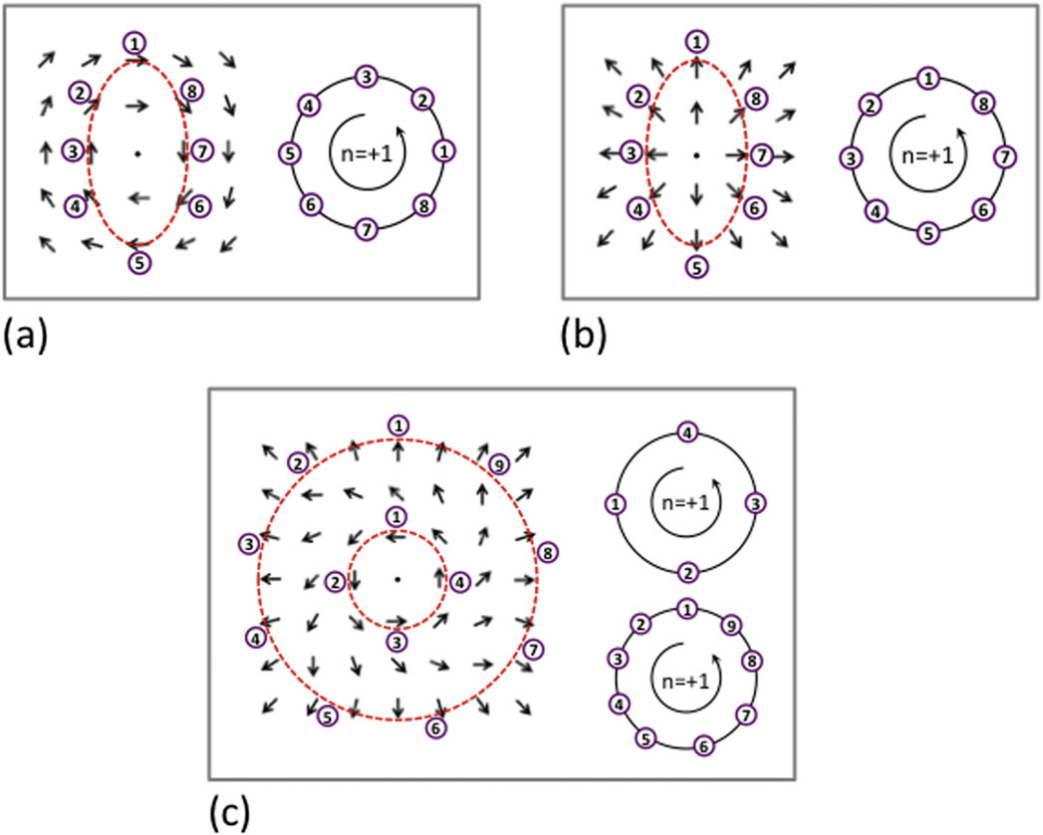
Schematic real space dipole patterns (left-hand side in each box) along with diagrammatic constructions used to determine winding numbers (right-hand side in each box). The development of the winding number diagrams involves consideration of a closed loop path in the real space dipole pattern (in each case considered, the path is marked with a red dashed line). On traversing the path in an anticlockwise sense, the orientations of dipoles successively encountered are recorded as poles on a circle, where the pole represents the intersection of the circle circumference and the dipole vector direction. After traversing the complete loop, the winding number is determined as the number of times the dipole vector poles rotate around the circle in an anticlockwise sense. As can be seen, rotating dipole patterns (a) and diverging dipoles (b) both have winding numbers of +1. Since they share the same winding number, one pattern can seamlessly transform to the other without the introduction of additional topological defects (c).

One of the early reviews on topological defects in crystals was that of Mermin in 1979 [1], which showed the difference between vertex domains (simple crossings of three or more domain walls) and vortex domains. The latter require a curl of polarization. A few authors use vertex and vortex interchangeably, which we argue against. Some claim that one can discriminate vertex from vortex by winding numbers. This is not true, as illustrated in [1]; all three polarization geometries in that review have winding number +1, but one is a pure divergence; one is a pure curl; and one has both. See also figure 1 below. A more complex set of ferroelectric winding numbers from −1 to +3 is discussed in detail elsewhere [2, 3].

## 2. Dynamics of shape-induced phase transitions in domain patterns

### 2.1. Stress and strain in nano-crystals

It was shown by some of us [4] that the Landau–Lifshitz–Kittel Law, which predicts domain stripe width w proportional to the square root of sample thickness *D*, is well satisfied for *D* all the way from nm to mm. However, this is true theoretically and experimentally only for specimens of infinite lateral width—or more precisely, of samples for which the lateral width *Y* ≫ *D* (hence the aspect ratio is essentially infinite). The present interest in nano-devices made it desirable to extend such theories to incorporate finite diameters [5].

### 2.2. Clock models, Potts models, hexagons, hexatics and Kosterlitz–Thouless melting

Early work [6] showed that vertex domains in a ferroelectric could be stable as fourfold vertices or as adjacent pairs of threefold vertices (vortex–antivortex pairs), but not both. For a three state Potts model (extension of scalar Ising model to three dimensions), the fourfold vertex is unstable and separates into adjacent threefold pairs. In contrast, for the three state clock model (vector Potts model), the threefold vertices are unstable and will coalesce into a single fourfold closure domain vertex. Both tungsten bronzes [7] and perovskites [8] favor the three-state Potts model.

One might ask whether hexagonal vertex structures in ferroelectric polarization exist in thin films, and if so, whether they imply hexatic phases requisite for two-dimensional melting. The answer is that they do exist [9–11] but as kinetically driven nonequilibrium states. And recent work has shown that Potts models with *n* > 5 are required to generate hexatic Kosterlitz–Thouless melting in two dimensions [12].

### 2.3. Stress in ellipses and rectangles

It has also been of interest to specify not only the diameter/thickness aspect ratio of ferroelectrics, but to include also their length/width ratio, since many nano-devices, such as memory elements in the Samsung 16 Gbit ferroelectric random access memory, are rectangular rather than circular or square. Work in 2011 showed [13] that rectangular ferroelectric films exhibited changes in the location of domain vertex centers related to the ratio *b*/*a* of their sides. A numerical simulation confirmed that this was the low-energy configuration, and the order parameter (vertex displacement) was identified, but the conjugate force remained enigmatic [13]. Showed that the position of closure domain vertices in ferroelectric thin films shifted off-center in rectangular specimens *a* × *b* in dimension by an amount related to the aspect ratio *b*/*a*. This was found to be compatible with geometry minimization of energy, but the conjugate field was not identified. That is, the kinematics were detailed, but the driving force was ambiguous (kinetics without dynamics).

In the present work we show that this phenomenon is compatible with stress, including hoop stress, which has usually been ignored in nano-physics [14, 15] but is well known in architecture [16].

**Figure 2.**
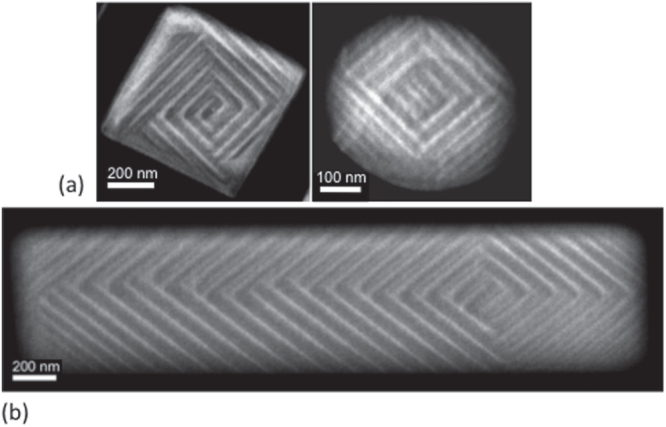
Scanning transmission electron microscopy (STEM) images of the ferroelastic domains that form, on cooling through the Curie temperature, in patterned single crystal BaTiO_3_ shapes. The vertex point, where domain bundles meet, moves from approximately the geometric center in squares and circles (a) to strongly off-center in oblongs (b).

Before proceeding to the nano-domain data in rectangular ferroelectric crystallites, it is pedagogically simpler to begin with disks, ellipses, and squares. Electron micrographs (STEM) of barium titanate are shown in figure 2.

In a circular closure domain, as illustrated in [17] the vertex usually lies at the center of the disk. This is also true in square specimens, as shown in figure 4 [18].

If we have an ellipse instead of a circular domain array, the vertex moves to one of the ellipse focal points. Recall that this shift is *f*/2, where

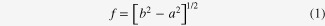
and *b* and *a* are the major and minor axes.

**Figure 3.**
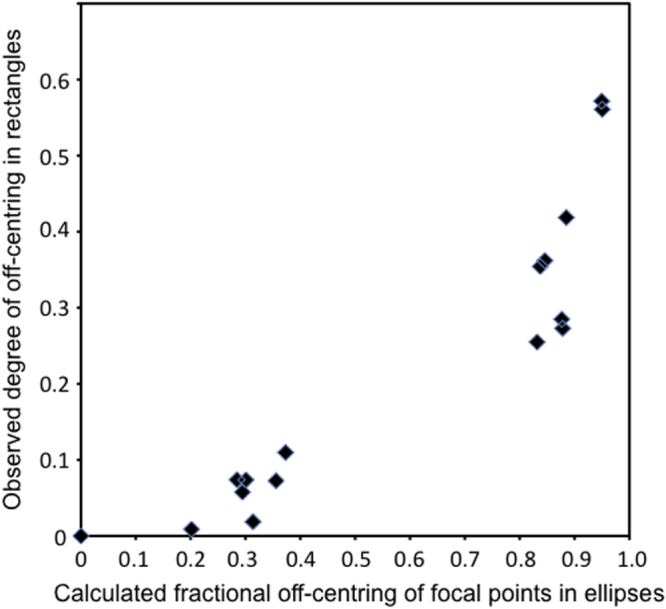
Plot of the degree to which domain vertex junctions move off-center as a function of the expected degree of off-centering if vertex positions were positioned at one of the foci in an ellipse with major and minor axes equivalent to the length and width of the rectangular BaTiO_3_ bars. While there is clearly a correlation, the relationship is nonlinear.

In a rectangle the sides of length *b* and *a* are analogous to the major and minor axes of an ellipse, and as shown in figure 3, the vertex loci shift strongly off-center.

**Figure 4.**
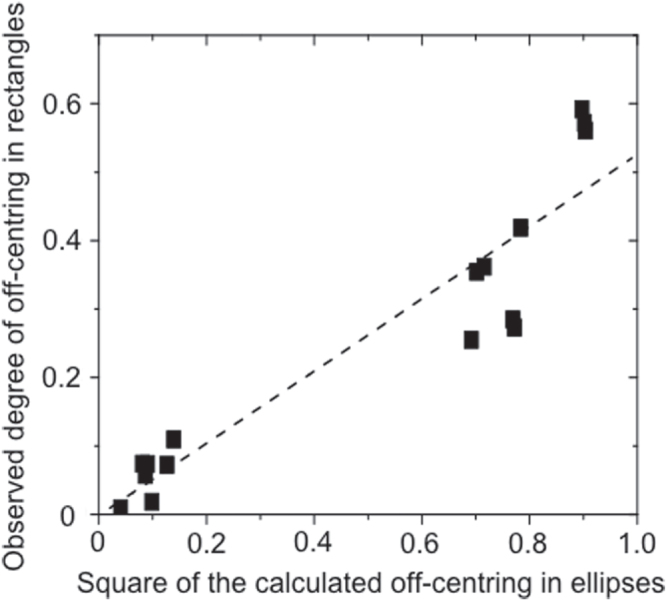
Plot of the degree to which domain vertex junctions move off-center as a function of the square of the off-centering associated with the foci of ellipses with major and minor axes given by the length and width of the rectangular BaTiO_3_ bars. While the plot is reasonably linear, it has a gradient of approximately 0.5 (as opposed to 1).

**Figure 5.**
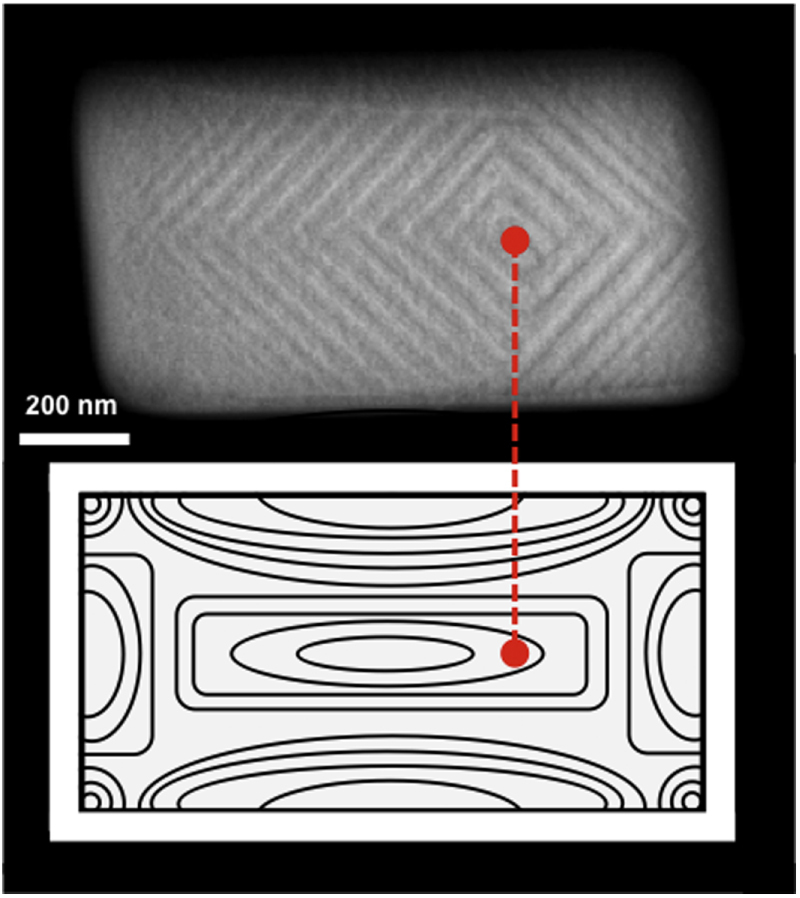
Direct comparison between the domain pattern developed in BaTiO_3_ rectangular dots with length/width ratio 2:1 and the torsional stress distribution in a twisted bar of the same aspect ratio, adapted from the model calculated by Francu *et al* [19]. While the domain pattern vertex junction does not obviously sit at a stress minimum, it is conceivable that it could if the stress pattern developed were in a different mode of distortion.

The formula for maximum stress in two-dimensional rectangular plates has been solved analytically, but to our knowledge only for torsional strain along the long rectilinear axis; even in that case it is algebraically complicated, involving infinite series. However, for a few integer ratios of the side lengths *b*/*a*, results can be found in the literature [19]. In figure 4 we compare the shear stress minima calculated [19] with our experimentally domain pattern for *b*/*a* = 2. The results are similar but certainly not a match, and a quadratic relationship between the two loci is found empirically (figure 5). We have also compared not the equipotentials but the field contours of constant electric field *E*. However, the agreement between vertex loci for closure domains and extremal points in the field contours is also poor. This suggests that if the vertex sites are at extremal points in the strain field, the strain in our rectangles is definitely not torsional about an axis. Further cases are under study.

We do note that the trajectory of vertex collisions in ferroelectrics reported [8] with its abrupt 90° turn follows the path of constant deformation (strain) calculated [19], suggesting an explanation for such motion that transcends the atomic-scale lattice structure and dominated by macroscopic or mesoscopic boundary conditions.

Thus the enigmatic driving force for shape-driven transitions in domain patterns, particularly of the off-centering of closure domain vertices, has been given a plausible quantitative explanation in terms of the aspect ratio *b*/*a* for rectangular specimens, but no quantitative agreement is found for a specific (torsional) strain.

The agreement shown above implies that uniaxial stress is only a vague qualitative mechanism (conjugate field) for the off-centering of domain vertices in ferroelectrics.

## 3. Hoop stress

**Figure 6.**
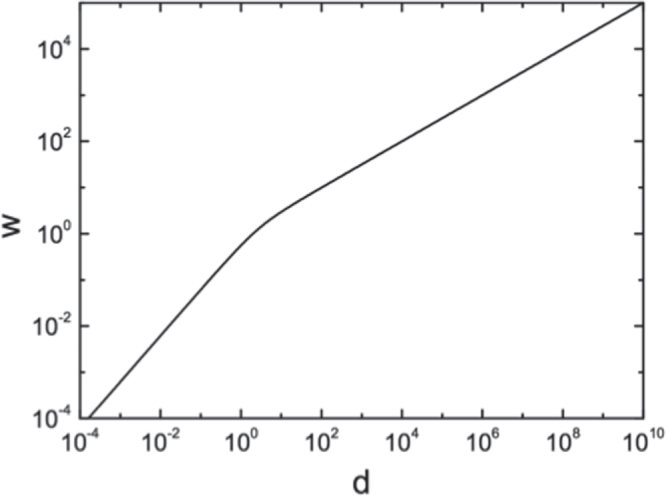
Prediction of the scaling behavior of ferroelectric domain width (*w*) with thickness (*d*) according to a Landau–Lifshitz–Kittel scaling law modified by the addition of hoop stress (equation (2) in the text). The hoop stress term dominates at low thickness values, generating a purely linear dependence.

As discussed above, the Landau–Lifshitz–Kittel Law for stripe domain width is based upon the balance of axial stress and depolarization fields. If there were only axial stress, materials would usually exhibit single-domain states. However, breaking up into narrow domains saves depolarization energy at the surfaces. But such domains increase wall energy. In their calculations Landau and Kittel ignored ‘hoop stress’ (or cylinder stress), because they were modeling parallel-plate capacitors of infinite lateral area. Of course, modern physics and device engineering emphasize nano-crystals of small lateral size (the crystals studied in [20] are only 8 nm in diameter). This finite size implies that ‘hoop stress’ (or cylinder stress) is not negligible. This is well known to architects and mechanical engineers. This is an azimuthal or tangential stress that increases the circumference of a ring or disk. The important thing is that it varies not as reciprocal area of the base, but as the circumference. Putting this extra term in 1/*r* into the Landau free energy, together with the original 1/*r*-squared stress term gives the result shown in figure 6 and equation (2) below

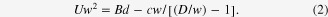


**Figure 7.**
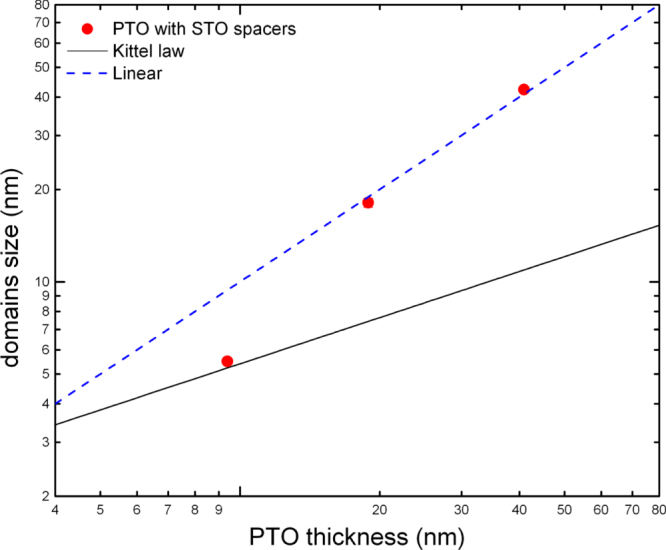
Domain size (determined by Fourier analysis of real space piezoresponse force microscopy images of PbTiO_3_ thin films grown onto SrRuO_3_ lower electrodes between insulating SrTiO_3_ spacer layers) as a function of ferroelectric thickness by Lichtensteiger *et al* [21, 22].

For small *r* (nano-disks) the dependence of w upon thickness *D* is linear (figure 6). This seems to be confirmed (figure 7) in very new data on PbTiO_3_ from [21, 22]; see also [23].

## 4. Creep and domain wall velocities

The domain wall velocities measured are ca. 1 nm s^−1^ for small fields *E*. This agrees with the creep velocities measured earlier [24] and seems characteristic of most oxide perovskites.

## 5. Quantum critical point in a uniaxial ferroelectric

We have examined the dielectric constant and loss in highly uniaxial ferroelectrics tris-sarcosine calcium bromide and TSCB substituted with chlorine or iodine. Unlike the pseudocubic SrTiO_3_ or KTaO_3_, these have effective dimensionality *d*_eff_ = 5 and are predicted [25] to exhibit dielectric constant *∊*′(*T*) varying as ${{T}^{-3}}$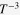 rather than the ${{T}^{-2}}$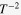 observed in strontium titanate and potassium tantalate. However, their polarizations are ultra-weak (Curie constant ca. *C* = 25–45 K compared with 50 000 K in BaTiO_3_), resulting in an experimental dependence of ${{T}^{-2.0}}$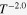 from ca. 1 to 50 K. Fits to the Barrett equation are discussed elsewhere.

### 5.1. Theory

#### 5.1.1. General QCPs: pseudocubic perovskites

**Figure 8.**
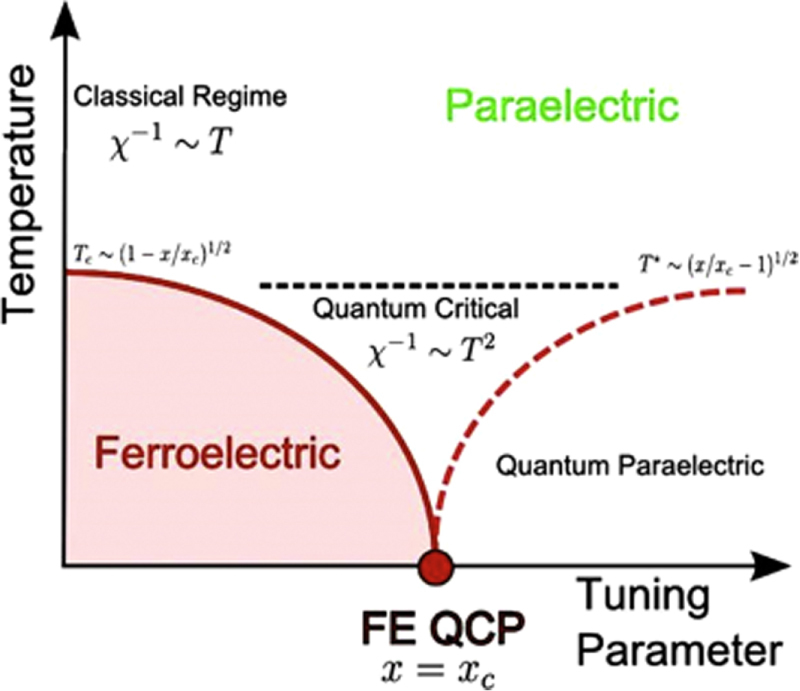
Schematic phase diagram for a quantum critical point. The tuning parameter *x* can be pressure, percentage atomic substitution, etc. The equations shown in the figure are for the case for a three dimensional system with a multi-axial polarization.

The general phase diagram of a quantum critical point [26] n a ferroelectric is schematically illustrated in figure 8.

The excitations around the critical point for a second-order displacive ferroelectric phase transition are propagating soft transverse optic phonons of very long wavelength (*q* = 0), which at the critical temperature are gapless. The long range dipole term is Coulombic in origin rather than relativistic as in the magnetic case, and is thus orders of magnitude stronger.In a multi-axial displacive ferroelectric the transverse optical mode exhibits dispersion of form ${{\Omega }^{2}}_{q}$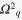 = *Δ*^2^ + *v*^2^*q*^2^ with *Δ* going to zero at the critical temperature *T* = *T*_c_. The parameter *v* is the speed of sound of the phonons when the gap *Δ* vanishes. The longitudinal optical mode frequencies remain finite at the critical point (but in tris-sarcosine calcium chloride—see below—become very small also at approximately 2 cm^−1^ (60 GHz) at exactly *T*_c_). In the self-consistent field model, which has been shown to be quantitatively applicable without any adjustable parameters in a number of ferroelectric systems [26, 27], the correction to the dielectric susceptibility due to quantum critical fluctuations is

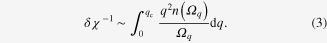
Here *n*(*Ω*_*q*_) is the Bose population at the transverse optical phonon frequency, and *q*_c_ is a cut-off wavevector typically taken to be the Brillouin zone boundary. In the classical regime for a material with or without a finite Curie temperature, and well away from the quantum critical point, the model predicts a Curie–Weiss like susceptibility *χ*^−1^ proportional to (*T* − *T*_c_). Close to the quantum critical point the equations become independent of the cut-off wavevector and lead to a temperature dependence which may be expressed in closed form as follows

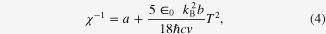

where *a*, *b* and *c* are the parameters of the Ginzburg–Landau free-energy expansion in the polarization *P* at zero temperature, i.e. *f* = (*a*/2)*P*^2^ + (*b*/4)*P*^4^ + (*c*/2) (*∇P*)^2^, *∈*_0_ is the permittivity of free space, *k*_B_ the Boltzmann constant and *h* the Planck constant.

#### 5.1.2. Khmelnitskii uniaxial QCP [25]; non-perovskites

The situation is quite different for the case of a uniaxial system where due to the crystalline details the polarization is confined to vary along only the *z* direction. In this situation as well as the splitting of the frequencies between transverse and longitudinal optical phonon frequencies, the dispersion of the transverse phonons is modified as follows:

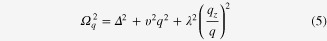


in which

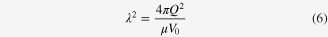
with *V*_0_ the volume of the unit cell; *Q*, the effective charge associated with the soft mode; and *μ*, the reciprocal mass of this normal mode. The non-analytic character of the last term in the right-hand side of equation (5) (it does not vanish as the wave-vector goes to zero) originates from the long-range character of dipole–dipole interactions. This is the case for both classical and QCPs. Noting that (*q*_*z*_/*q*)^2^ = cos^2^*θ* where *θ* is the angle between the *z* axis and the direction of wave propagation *q*, the integral in equation (3) now becomes

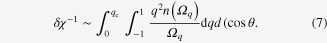


With an additional dimension and by an appropriate change of variables, the integral may be solved analytically close to the quantum critical point where *Δ* approaches zero. The result in this case is that the inverse susceptibility varies as the cube of the temperature [10]. Again this may be solved numerically giving the full temperature dependence of the susceptibility for uniaxial systems. Effectively the cos(*θ*) integral acts as one extra dimension:

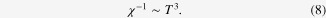


More generally, the inverse susceptibility defines an exponent *γ*, such that it varies as ${{T}^{\gamma }},$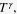 and *γ* is defined as *γ* = (*d* + *z* − 2)/*z*, (for pseudo-cubic symmetry *z* = 1, *d* = 3), so *γ* = 2 in pseudocubic perovskites. Here *z* is the dynamical exponent and is defined as the dependence of soft mode frequency upon wave vector near *q* = 0 in a displacive ferroelectric. In classical Newtonian physics the spatial fluctuations are separate from the temporal ones, so the temporal fluctuations do not enter the thermodynamics near *T*_c_; but in a quantum mechanical system these are intermixed because momentum and position do not commute.

**Figure 9.**
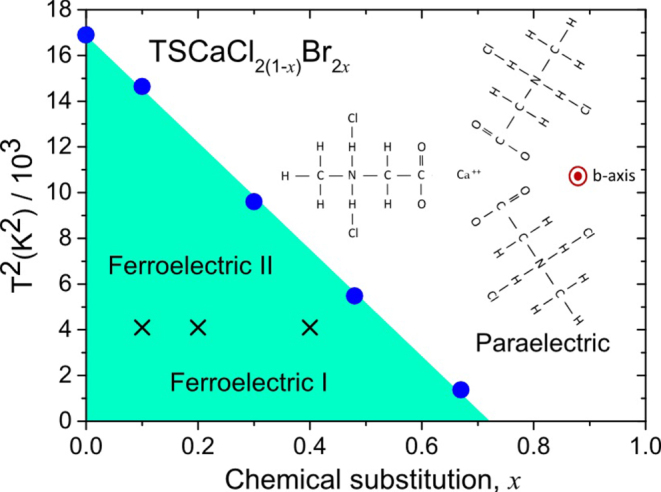
Phase diagram [27] of brominated tris-sarcosine calcium chloride (pseudo-hexagonal at all *T*, in contrast with SrTiO_3_, which is pseudo-cubic below *T* = 105 K).

Ferroelectricity in TSCC may be tuned to absolute zero using bromine substitution as shown in figure 9. The ferroelectric transition in TSCC is observed to be second order in all experiments carried out so far, an uncommon feature among ferroelectrics, which implies that the zero temperature transition is a QCP with quantum fluctuations.

#### 5.1.3. Reconciliation of the Khmelnitskii paradox

Generally speaking theories of quantum criticality in ferroelectrics assume that the spontaneous polarization *P*_s_(*T*) increases slowly as *T* approaches zero and is a constant for low *T*. However, there are a few exceptions. In comparison with ferromagnets, where the Curie constant in the Weiss theory is not an independent parameter but is given by

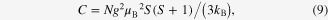
where *g*, *μ*, and *k* are the gyromagnetic ratio, Bohr magnerton, and Boltzmann constant, in ferroelectrics *C* is an empirical adjustable fitting constant of dimension *T*, given by

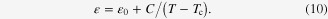


Some authors [28–30] have defined a ‘bare’ Curie constant

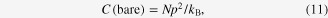
and term the ratio *C*/*C*(bare) the Rhodes–Whohlfarth ratio, in analogy with ferromagnets.

In order to describe Rochelle salt, early work [31] assumed a rotating rigid dipole model in which the polarizability

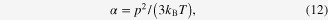
where each rotating dipole has polarization *p*.

The internal field is then given by

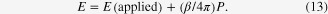


For the crude approximation of an array of point dipoles in a random lattice, *β* = (4/3) *π* and *C* = 3 *T*_c_. The data for *C*(*T*_c_) in TSCC/TSCB are show that *C* is indeed linear in *T*_c_, but it is ca. 1/9 *T*_c_, not 3 *T*_c_; hence the model of point dipoles in a random lattice gives the correct functional dependence but is quantitatively an order of magnitude off for the proportionality constant.

Hence in TSCC or its Br-isomorphs, *C* decreases to zero as bromination increases, down to the QCP. This renders the assumption of constant *P*(*T*) by Khmelnitskii and Larkin invalid and means TSCC/TSCB will behave as an isotropic material asymptotically as *T*_c_ approaches zero [30]. Consequently, and for reasons idiosyncratic to TSCC, this highly uniaxial material behaves like a pseudocubic perovskite near its QCP.

### 5.2. Electrocaloric effect

The electrocaloric effect is predicted to diverge as *T* approaches zero, due to the fact that from the Maxwell relations, in equilibrium, there is an indirect way of determining the temperature cooling *ΔT*

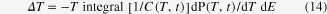
and this expression contains an integral over specific heat *C* that normally diverges faster as *T* goes to zero than can be compensated by its linear prefactor of *T*. However that assumes that polarization *P* is large and rather independent of *T* as *T* becomes very small (required by the Third Law of Thermodynamics); in the present case *P* is exceptionally small, and the numerical values of the electrocaloric effect are extremely small, even at low *T*.

Perhaps more interesting is the time dependence of these relations. The estimate in equation (14) is from the Maxwell relations and generally agrees within a factor of 2 or better with direct measurements of *ΔT* [32]. However, it is not exact. It is based upon equilibrium thermodynamics (dubious in the case of relaxors), and it ignores time dependences. *P*(*T*) actually depends upon time for real ferroelectrics due to relaxation processes [33], especially in oxide perovskites, where losses are often due to oxygen vacancies. After a voltage is applied *P*(*T*,*t*) decays with time t, on several different time scales, depending upon temperature *T*. Typically the long-term value of polarization *P* is ca. 50–80% of the short-term value. In some modern test equipment, the remanent polarization *P*_r_ at long times is denoted with a circumflex.

Thus if *ΔT* is measured on a time scale long compared with the relaxation time tau, care must be taken to use the proper value of *P* in equation (14), which may be only half that of the short-time measured electrical value from the *I*(*V*) hysteresis curve. This will generally lead to an overestimate of *ΔT* inferred from the ‘indirect’ method described by equation (14), and by as much as 30–50%. No published electrocaloric data include this time dependence of the Maxwell relations. To be more self-consistent, *ΔT* must be measured as a function of time *t* in applying equation (14), and the time scale must be commensurate with the relaxation time *P*(*T*,*t*) and also the time constant for the specific heat measurements *C*(*T*,*t*).

## 6. QCPs in multiferroics perovskite lead iron niobate, lead iron tantalate, and their single-phase mixtures with PZT

Dome-shaped phases in the graphs of *T* versus *x* in superconductors and magnets, where *x* is pressure, or magnetic field, or percentage concentration of some constituent element, are phenomena of great current interest. Interesting states of matter and unusual physical properties often are found near these phase boundaries. However, such dome-shaped phases are highly unusual if not unique in (*T*,*E*) phase diagrams in ferroelectrics. Here we examine the predicted presence of such domes in both lead iron niobate ( ${\rm PbF}{{{\rm e}}_{1/2}}{\rm N}{{{\rm b}}_{1/2}}{{{\rm O}}_{3}})$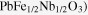 and lead iron tantalate ( ${\rm PbF}{{{\rm e}}_{1/2}}{\rm T}{{{\rm a}}_{1/2}}{{{\rm O}}_{3}})$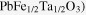 mixed into single chemical phase compounds with lead zirconate titanate (PbZr_0.47_Ti_0.53_O_3_) to produce high-temperature magnetoelectric multiferroics (lead iron niobate zirconate titanate PFNZT and lead iron tantalate zirconate titanate PFTZT). Each dome-shaped phase, according to Glinchuk *et al* [34] should exhibit a quantum critical point—a phase transition at *T* = 0—at ca. 5% and 20% Fe^+3^-ion B-site occupancy (four QCPs in all in the two materials PFNZT and PFTZT). The experimental data do not confirm such dome shapes in the phase diagram, but will be compared with the general theoretical predictions of others [35–37] and with experiments [38]. We have also measured the dielectric properties of a non-perovskite Ba-based M-type hexaferrite [(Ba,Sr)Fe_12_O_19_] down to 300 mK, with a view to understand its quantum critical point. We find two unexpected results: (1) below 5 K the dependence of dielectric constant *∊*′(*T*) is not monotonic and resembles that in SrTiO_3_ and KTaO_3_; we interpret this as arising from electrostrictive coupling of the soft mode to acoustic phonons; (2) the critical exponent gamma that describes the divergence of *∊*′(*T*) above 4 K describes a power-law dependence with *γ* = 3.0 ± 0.2, which differs significantly from the value 2.0 for quasi-cubic materials with *d* + 1 = 4 but agrees with Khmelnitskii’s theory for anisotropic uniaxial ferroelectrics with *d* + 1 = 5 and *γ* = 3. A model for this will be presented elsewhere that is an alternative to the phenomenological Barrett equation, related to [24].

Most interesting is the non-monotonic dip in reciprocal dielectric constant versus temperature 1/*∊*′(*T*) as *T* goes below ca. 4 K. This is due to electrostrictive coupling to acoustic phonons [26, 27], and it shows that this behavior is rather universal and not restricted to perovskites.

## 7. Room-temperature magnetoelectric and multiferroic GaFeO_3_

Although gallium ferrite has an ABO_3_ formula, it is not a perovskite but rather an orthoferrite. Its apparent multiferroic behavior has been puzzling [39]. We have examined the orthoferrite GaFeO_3_ by atomic force microscopy (AFM), dielectric techniques, specific heat, and resonant ultrasonic techniques, comparing bulk samples with thin films 10–200 nm in thickness. The results show that this material is ferromagnetic and pyroelectric at room temperature (sufficient for magnetoelectric coupling of form PM) and also ferroelectric. This is not compatible with the earlier conclusion [40, 41] that the barrier between P2_1_/a states via a centered Pnma state of 1.1 eV is too high for switching without breakdown.

A recent series of papers has inferred [32] multiferroic behavior at room temperature for gallium ferrite, but the ferroelectric hysteresis loops were somewhat unconvincing for bulk while clear for some very thin films. Meanwhile studies of thin films of this material [40, 41] concluded that the coercive field at room temperature exceeds the breakdown field, so that the material is pyroelectric with a barrier >1.0 eV for switching, but not ferroelectric. Pyroelectricity is a symmetry property of crystals, whereas ferroelectricity is a practical engineering definition: If it cannot be switched via an applied electric field, it is not ferroelectric (‘ferroelectric metals’ not withstanding).

In our present studies we are comparing bulk and thin-film gallium orthoferrite to try to resolve this paradox. We note that bulk samples from India exhibit no stripe domains or AFM switching, whereas thin films do. We observe good ferroelectric hysteresis loops in 50, 100 and 200 nm GaFeO_3_ films up to *T* = 400 K, above which electrical conduction is too high. Typically *P*_r_ = 20 *μ*C cm^−2^ and *E*_c_ = 30 kV cm^−1^. We further note that multiferroic behavior has been measured in SmFeO_3_, which had been thought to be an orthoferrite structure also, with any magnetoelectric coupling forbidden [42–44], so orthoferrites like perovskites merit further study.

## 8. Thermal expansion and extrinsic critical exponents

The thermal expansion near QCPs is predicted to vary as *T* (not as ${{T}^{3}})$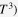 but this has not been measured. Our work to be reported elsewhere tests this hypothesis for oxygen-18 isotope-enriched SrTiO_3_, TSCC:Br, and BaFe_12_O_19_.

## 9. Contrast with magnetic walls

Textbooks sometimes assume that ferroelectric wall dynamics is analogous to that of magnetic walls. This is not true in several respects: First, magnetic walls can easily be driven supersonic, where they emit coherent acoustic phonons at a bow-wave angle similar to that in Cherenkov radiation, whereas ferroelectric walls cannot be (the shock wave would destroy the crystal), and evidence apparently to the contrary in early textbooks arose from the false assumption that all domains nucleated at the cathode and moved to the anode, giving a velocity *v* = thickness/time; of course many of the domains nucleated midway between the electrodes. Second, magnetic walls obey the Landau–Lifshitz–Gilbert equations, which are first-order in time. Such equations require instant stopping when the external field *H* is turned off—no momentum and no coasting. In contrast, ferroelectric walls obey Newton’s Second Law, which is second order in time and requires momentum and coasting after the field E is terminated (up to 50 *μ*m of coasting). Failure to recognize this coasting effect caused others to make claims about ferroelectric switching for which they ignored conservation of momentum [42–44].

## 10. Semiconducting properties

The two most important things about oxide ferroelectrics are: (1) in general they are not insulators. PbTiO_3_ has a band gap of approximately 2.96 eV, well below that of wide-gap III–V semiconductors such as GaN or II–VI’s such as ZnO. Therefore, especially in thin-film form, they are electrical conductors. Hence it is necessary for device development to understand their band structure, effective masses. Typically they are n-type with light and heavy electrons, and electron effective mass *m*∗ of order 5.5–6.5 *m*_e_, in contrast to the value *m*∗ = 1.0 *m*_e_ erroneously used by one group who inferred Fowler–Nordheim tunneling (also unlikely). [45–48]; although in principle the tunneling mass can differ from the effective mass, they must agree for tunnel barriers >2 nm [49] and do in careful experiments [50]. On metal electrodes with large Fermi energies such as Pt (work function *W* = 5.34 ± 0.02 eV) or Pd or Au, their large electron affinities usually produce small Schottky barrier heights, of order 0.8–1.2 eV. Because their mean free paths are small compared to the Schottky barrier widths, conduction is usually in the Simmons-limit of Schottky conduction, which yields current *J*(*E*) proportional to *E* times the usual exponent (*qV*/*kT*)^1/2^, where *V* is applied voltage. For low voltages the exponential is nearly unity, and *J* = *bE* results, giving the illusion of ohmic conduction (but unlike ohmic transport, insensitive to thickness and interface-limited); this aspect of Schottky barriers has been widely overlooked, most recently by a group at Cambridge, who concluded that linear *J*(*V*) currents in systems with large Schottky barriers were ohmic due to impurity banding [51]. (2) Six is bigger than three! Direct tunneling through oxide films is typically limited to thickness *D* < 6 nm, whereas ferroelectric polarization is stable for *D* > 2.4 nm. This gives a finite range of thickness for which ferroelectric tunnel junctions are attractive devices, as developed nicely at THALES. Unfortunately for three decades the scientific community was under the impression, based upon misleading work from IBM, Japan, and North Carolina that the minimum ferroelectric film thickness for stability was tens or even hundreds of nm. This error delayed progress for years.

## 11. Conclusions

The study of perovskites has recently explored situations where finite size effects and boundary conditions play a key role, and hence where the periodic cylindrical boundary conditions imposed in DFT theory fail. These situations are often kinetically limited and not in mechanical equilibrium; therefore Landau free energy theories fail, as do simple strain–equilibrium models such as the Landau–Lifshitz–Kittel Law.
